# Hydrophobia, Its Prevalence and Prevention*Read before the Medical Association of Georgia in Athens, April 20, 1910.

**Published:** 1910-05

**Authors:** Henry R. Slack

**Affiliations:** Superintendent of the LaGrange Sanatorium, LaGrange, Ga.


### HYDROPHOBIA, ITS PREVALENCE AND PREVENTION.*

HENRY R. SLACK, PH. M., M. D.,
*Superintendent of the LaGrange Sanatorium,*
*LaGrange, Ga.*

It is not the object of this paper to discuss the brilliant work of Pasteur in discovering the preventive treatment of rabies by animal experimentation nor the etiology and pathology of this disease, for you are all familiar with the literature on this subject. It is too extensive to attempt to condense into a paper of this length. I merely wish to call your attention to the rapid increase of this disease and point out the method for its prevention.

Ten years ago, in Atlanta, I presented a paper to this Association entitled "Hydrophobia, and the necessity for a Pasteur Institute in Georgia." This paper was fully discussed and the motion to appoint a committee with power to act prevailed. The work of this committee led to the establishment of the Georgia Pasteur Institute in 1900.

It was contended by quite a number of well informed physicians that there was not enough rabies in Georgia and neighboring states to justify the establishment of a Pasteur Institute, but Dr. Jas. N. Brawner took hold of it with energy and determined to make it a success.

We treated during the first year only 22 cases, the second 43, the third year 85, fourth year 87, fifth year 108, sixth year 155,

*Read before the Medical Association of Georgia in Athens, April 20, 1910.

seventh year 206, eighth year 276, and the ninth year 68. Meantime the legislature, on motion of Dr. L. G. Hardman, appropriated money for the State Board of Health to established a Pasteur department which was opened in June, 1908, under the able management of Dr. H. F. Harris and Dr. Edgar Paullin, and from then until December 31st, they treated 216, in 1909 they treated 418, and up to April 15th of this year, 149 cases. Thus it will be seen that there were 1830 persons who have received Pasteur treatment in the past nine years in Georgia, and this despite the fact that nearly all the Southern States have established Pasteur laboratories for the free treatment of their citizens.

Of the 1830 persons treated, only 6 developed the disease after completion of the immunity period. which is less than one-third of one per cent., while 25 per cent. of those bitten who failed to take the treatment died. This shows a saving of over 400 lives by Pasteur treatment in Georgia alone in the last ten years.

When you consider the fact that for four years, (from 1900 to 1904), we only treated 237 patients and that 44 of them came from west of the Mississippi River, 32 from Alabama, 24 from South Carolina, 118 (less than half from Georgia) and that 489 were treated in Georgia in 1908 and 486 in 1909, after these other states had established Pasteur laboratories, the increase in rabies is appalling. The average for the first four years of this decade was 59.5 while for the last two years it is 487.5 or an increase of over 837 per cent in less than ten years. This certainly is enough to make us pause and think what we must do to prevent the rapid spread of this most awful of all diseases.

We are all more or less familiar with Death when he comes in "Consumption's ghastly form, the earthffuake shock and ocean storm," but in hydrophobia he is even more terrible. Now all this anxiety, suffering and death can be prevented by a very simple method.

Muzzle all dogs and establish a six months' quarantine. This is not a theory, but a fact that has been demonstrated in England, Denmark, Sweden and Norway. In the two latter countries there has not been a case of rabies in over fifty years. England's experience with the muzzling law demonstrates its efficacy most clearly. In 1889 there were 312 dogs that died with rabies and thirty human beings. The muzzling law was passed and rigidly enforced, so that in 1892 (three years) only 38 dogs and 6 people died with rabies. The "dog lovers" at this time appealed to the authorities to remove the "cruel muzzle" and they consented with the result that during the next five years 1602 dogs and 51 people died of the most agonizing disease known to medicine. Despite this awful lesson these same "humanitarians" came again in 1899 with another petition signed by 50,000 of them begging to be relieved of the annoying muzzle, but the authorities had learned a lesson and remained obdurate, with the result that in 1905 no case of rabies occurred in England and there has been none since in man or beast.

What has been accomplished in England, Sweden, Denmark and Norway can be done in the United States, and we must see to it that Georgia takes the lead in this matter and that our next legislature passes a muzzling law. The dog tax is a failure, a fallacy and a fraud as at present enforced.

I hope the Association will instruct the Legislative Committee to work for this law, and let us stamp out hydrophobia from the Empire State of the South.

